# Experimental Oral Transmission of Atypical Scrapie to Sheep

**DOI:** 10.3201/eid1705.101654

**Published:** 2011-05

**Authors:** Marion M. Simmons, S. Jo Moore, Timm Konold, Lisa Thurston, Linda A. Terry, Leigh Thorne, Richard Lockey, Chris Vickery, Stephen A.C. Hawkins, Melanie J. Chaplin, John Spiropoulos

**Affiliations:** Author affiliation: Veterinary Laboratories Agency–Weybridge, Addlestone, UK

**Keywords:** sheep, scrapie agent, atypical, oral administration, bioassay, infectivity, alimentary system, prions and related diseases, research

## Abstract

To investigate the possibility of oral transmission of atypical scrapie in sheep and determine the distribution of infectivity in the animals’ peripheral tissues, we challenged neonatal lambs orally with atypical scrapie; they were then killed at 12 or 24 months. Screening test results were negative for disease-specific prion protein in all but 2 recipients; they had positive results for examination of brain, but negative for peripheral tissues. Infectivity of brain, distal ileum, and spleen from all animals was assessed in mouse bioassays; positive results were obtained from tissues that had negative results on screening. These findings demonstrate that atypical scrapie can be transmitted orally and indicate that it has the potential for natural transmission and iatrogenic spread through animal feed. Detection of infectivity in tissues negative by current surveillance methods indicates that diagnostic sensitivity is suboptimal for atypical scrapie, and potentially infectious material may be able to pass into the human food chain.

## Abstract 2

Such transmission results in peripheral tissue infectivity that is not detectable by current surveillance screening methods.

Since the discovery of atypical scrapie (*1*) and its subsequent identification, mostly through active surveillance, in several countries (some with no previous history of transmissible spongiform encephalopathies [TSEs]) (*2**,**3*) such as New Zealand (*4*) and Australia (*5*), scientists have debated whether this form of TSE is in fact spontaneous or acquired (*3**,**4**,**6**–**8*) rather than contagious. The epidemiologic studies that have been undertaken suggest that atypical scrapie does not appear be transmitted between animals in the field situation (*7**,**8*). Although the routes by which natural transmission occurs have never been fully established for TSEs, it is widely accepted that ingestion of infective material, i.e., the oral route, is a key component in some TSEs, e.g., kuru (*9*), variant Creutzfeldt-Jakob disease (*10*), bovine spongiform encephalopathy (*11*), and transmissible mink encephalopathy (*12*).

Within the sheep population, susceptibility to particular strains of TSE has been shown to be heavily affected by polymorphisms of the prion protein gene of the sheep (*13**–**18*). The successful transmission of atypical scrapie to sheep after intracerebral inoculation has been previously reported for sheep of 1 genotype (A_136_H_154_Q_171_/A_136_H_154_Q_171_) (*19**,**20*), and challenges in other homologous and heterologous genotype combinations are ongoing. However, successful intracerebral transmission of a particular TSE agent in a particular species does not necessarily indicate susceptibility by the oral route (*21*).

The tissue distribution of infectivity or disease-specific prion protein (PrP^Sc^) in bovine spongiform encephalopathy in sheep has led to extensive public health control measures based on the known pathogenesis and distribution of PrP^Sc^ in edible tissues, and their removal from carcasses of animals over a certain age. Classic scrapie may also show the widespread accumulation of PrP^Sc^ in peripheral tissues. Although early studies of atypical scrapie did not show PrP^Sc^ or infectivity outside the brain, recent data indicate that peripheral tissues from naturally infected animals can harbor infectivity either in the presence or absence of PrP^Sc^ (*22*). However, whether this infectivity is established before or after the agent has propagated in the central nervous system is unknown.

The first aim of the current study was to examine the distribution of infectivity in peripheral tissues in animals at and beyond the cutoff point for the current meat hygiene regulations of the European Commission (i.e., 12 months of age). The second aim was to investigate the potential for oral transmission of atypical scrapie.

## Methods

### Recipient Animals

Six Cheviot lambs with PrP genotype AHQ/AHQ and 6 Poll Dorset lambs with the genotype ARR/ARR were sourced from the New Zealand–derived flock owned by the UK Department of Food, Environment and Rural Affairs (*3*), and transported in utero with their dams to the Veterinary Laboratories Agency–Weybridge before lambing. All procedures involving animals were carried out in accordance with the UK Animal (Scientific Procedures) Act 1986, under license from the UK Government Home Office.

### Experimental Challenge

Within 24 hours of birth, during February–March 2008, each lamb was challenged orally with 2.5 g of brain homogenate prepared from animals with naturally acquired cases of atypical scrapie of the same genotypes, and again with a further 2.5 g of the same homogenate 14 days later. Homogenate was delivered through a syringe to the oropharynx (*23*). The lambs were kept with their dams and separate from each other until they were weaned at 9–11 weeks of age; after weaning, they were housed in groups according to genotype.

### Donor Animals

#### Inoculum ARRa

This animal was identified through passive surveillance in 2004. The sample was submitted before ELISA screening was used routinely for passive surveillance, and no inoculum remained for further biochemical characterization after the challenge of 5 lambs. Results of immunolabeling in the medulla, cerebellum, and thalamus, and Western blot on the medulla were consistent with atypical scrapie (*24*) (data not shown).

#### Inoculum ARRb

This animal was identified through active surveillance (fallen stock). ELISA (Bio-Rad Laboratories, Marnes-la-Coquette, France) of this inoculum gave optical density (OD) values of 1.554 and 0.761. The OD of the initial diagnostic screening sample was 1.074. Results of Western blot and immunolabeling throughout the neuraxis were consistent with atypical scrapie (data not shown).

#### Inoculum AHQ

This animal was identified through active surveillance (fallen stock). ELISA (Bio-Rad Laboratories) of inoculum prepared from this animal gave a positive OD reading of 2.272. The initial diagnostic screening sample gave an ELISA OD of 0.948. Western blot and immunolabelling results were consistent with atypical scrapie, but with a preponderance of white matter staining and substantially less neuropil staining than has been seen in other AHQ atypical cases (data not shown).

### Clinical Monitoring

All animals were monitored daily during routine husbandry procedures and monthly during blood sampling from 8 months post inoculation. Within 12 days of the cull date for each sheep, a clinical and neurologic examination was carried out, including cranial nerve assessment and testing of response to scratching of the back according to published protocols (*23*). On the basis of clinical signs, the animal’s clinical TSE status was categorized as follows: normal (no apparent signs of scrapie), inconclusive with regards to scrapie (e.g., impaired menace response, minor wool loss), and suspected scrapie (combination of abnormalities in behavior, sensation, or movement), as has been described for goats (*25*).

### Cull Schedule and Sampling

Three animals of each genotype were selected at random and culled at 12 months of age, and the remaining 3 were culled at 24 months of age. Animals were euthanized by using quinalbarbitone sodium (Somulose–Dechra Veterinary Products, Shrewsbury, UK), and a variety of samples of neural and non-neural tissues (Table A1) were obtained by using aseptic techniques and either placed into 10% formal saline (neural tissues), 10% buffered formalin (non-neural tissues) or frozen and held at –80°C for subsequent examination by immunohistochemical (IHC) testing, ELISA, or both (Table), depending on the size and nature of the sample. Some samples were collected for examination in the event of a positive result in the samples chosen for initial screening.

### Immunohistochemical Testing

Fixed samples were processed into paraffin wax, sectioned, and stained with hematoxylin and eosin as described (*26*). IHC labeling to detect PrP^Sc^ was performed as previously described (*19*) by using mouse monoclonal antibody 2G11 (Institut Pourquier, Montpellier, France), raised against ovine PrP peptide sequence 146-R154 R171–182. IHC profiles were created by using a standard subjective method as previously described (*24*) in which the type of PrP^Sc^ immunolabeling is assessed in a standard range of precise neuro-anatomical areas. Tissues from the lympho-reticular system of each challenged animal were examined by IHC as described above.

### ELISA

All tissues were analyzed by using the TeSeE kit (Bio-Rad Laboratories) for TSE detection in sheep and goats, according to the manufacturer’s instructions. The cutoff value was calculated as the average absorbance reading of the negative control values plus a value of 0.14 absorbance units. To directly measure PrP^Sc^ content in the samples that were used to inoculate the recipient animals, the inocula were prepared for the Bio-Rad protocol before analysis to account for variation in tissue and buffer content. Samples were centrifuged at high speed to concentrate insoluble material. The weight of the pellet was calculated, and then it was resuspended in homogenization buffer to prepare a 20% wt/vol homogenate and continued as described from the ribolysation stage. Pellets that contained insufficient weight to prepare 250 μL of 20% wt/vol homogenate were supplemented by using brain homogenate or relevant tissue prepared from sheep that had not been exposed to scrapie (control reference material) before testing.

### Western Blot

Positive, fresh brain samples were subjected to the TeSeE Universal Western blot (Bio-Rad Laboratories, catalog no. 355 1169) as described (*19*). Molecular mass markers were included at either end of the gel. A single lane each of samples from animals with known UK classic scrapie, known UK bovine BSE, and a known UK atypical scrapie were included for profile comparisons.

### Mouse Bioassay

Brain areas from animals positive for PrP^Sc^ were assayed in mice to assess the stability of agent following passage. In addition, bioassay of 3 additional tissues (cerebellum, spleen, and distal ileum) has also been initiated from every challenged sheep whether all tests were negative or not (Table A1). Samples from the donor and experimental recipient sheep were treated in the same way. Tissue homogenate (10%) was prepared wt/vol in normal saline, screened for microbiologic sterility by using standard methods, treated using ampicillin and gentamicin if contamination was identified, and rechecked before use.

Panels of 10 transgenic mice overexpressing ovine prion protein gene (Tg338 [*27*]) were inoculated intracerebrally with 20 μL and, when sufficient inoculum was available, intraperitoneally with 100 μL of homogenate. Mice were monitored weekly and were killed when they had shown clinical signs on 2 of 3 consecutive monitoring days or had reached their natural lifespan. Brains were then fixed and processed, and lesion profiles were produced as described in detail elsewhere (*28*).

## Results

Full details of genotype, clinical status, kill time points, and test results for each animal are shown in the online Table A1. A proportion of the mouse infectivity bioassays are incomplete, as indicated in the text below, so some tissues that are currently considered negative for PrP^Sc^ by bioassay may change status at a later date.

### Cull at 12 Months

Of the 6 animals (3 ARR/ARR and 3 AHQ/AHQ) killed after 12 months, 5 were clinically healthy at the time of culling. Animal 3 appeared nervous during handling, although its behavior at previous blood sampling sessions had been unremarkable, and it displayed a bilaterally absent menace response (inconclusive results with regards to scrapie).

**Figure 1:**
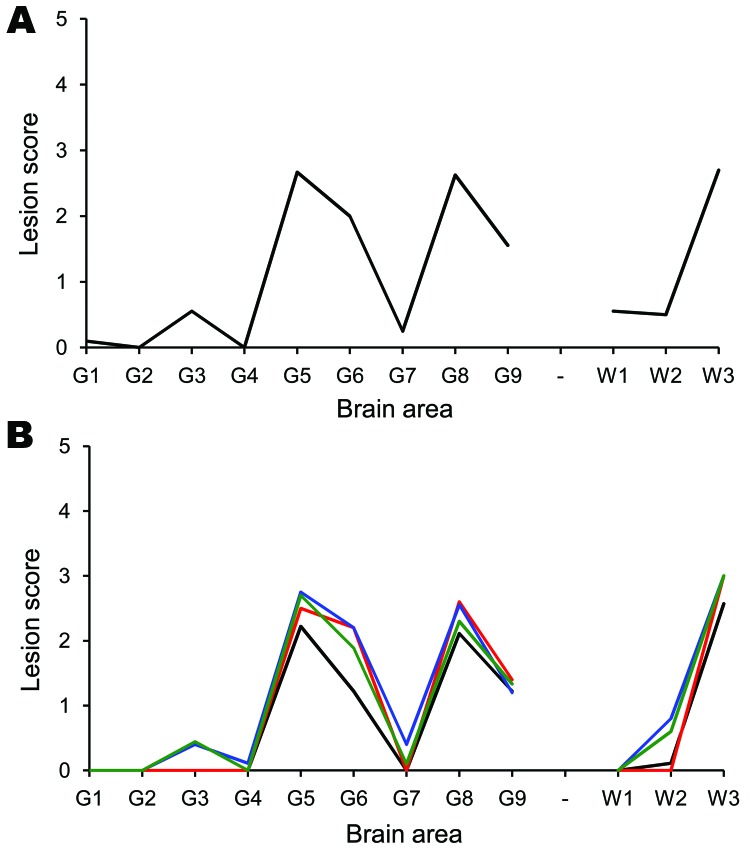
Tg338 mouse vacuolation lesion profiles of mice infected with scrapie. Only clinically affected mice were considered when generating the lesion profiles. Number in parentheses indicates the mean incubation period of the mice, which contributed to the lesion profile. A) The donor AHQ/AHQ sheep (177 ± 3; n = 10). This profile is compatible with that obtained from other naturally-occurring cases of atypical scrapie (*19*,*20*). B) Recipient sheep brain and distal ileum. Cerebellum from animal 12 (179 ± 12; n = 6/10) is indicated in red. Basal ganglion from animal 11 (193 ± 7; n = 10/10) is indicated in blue. Hippocampus from animal 11 (198 ± 11; n = 10/10) is indicated in green. Distal ileum from animal 9 (247 ± 23; n = 9/9) is indicated in black.

None of the sheep exhibited PrP^Sc^ in the tissues examined. Bioassays in mice of the cerebellum, spleen, and distal ileum from each animal are ongoing. To date, infectivity has been demonstrated in the cerebellum of animal 1 and the distal ileum of animals 8 and 9. Only the bioassay from animal 9 has been completed, with 9 mice succumbing to disease (Figure 1). A proportion of the mice used in the assays of samples from animals 1 and 8are still alive.

### Cull at 24 Months

Of the 6 sheep culled at 24 months post inoculation, 3 appeared clinically normal. Animal 6 displayed alopecia suggestive of pruritus although no pruritic behavior was observed (inconclusive with regards to scrapie). Animal 10 appeared nervous when it was approached and handled, which had not been observed on previous blood sampling sessions, and displayed a wide-based hind limb posture (inconclusive with regards to scrapie). Animal 12 appeared nervous with a fine head tremor during handling, had a wide-based hind limb posture, and was ataxic with uncoordinated jumps, swaying, and loss of balance (Video). At blood sampling 2 months earlier, the sheep had been observed circling clockwise when left alone, which was not seen at the final examination (suspected scrapie). None of the sheep had a positive scratch test, and none of these 6 sheep had lost weight before cull.

**Video:**
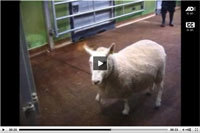
This sheep has a tendency to sway excessively, which is particularly noticeable when it stops suddenly. Note the broad-based posture of the hind limbs when the sheep stands facing the gate.

Direct Link: http://streaming.cdc.gov/vod.php?id=c3948e8d12a7c29aec0d7906fc8ef75920110429152854419

**Figure 2:**
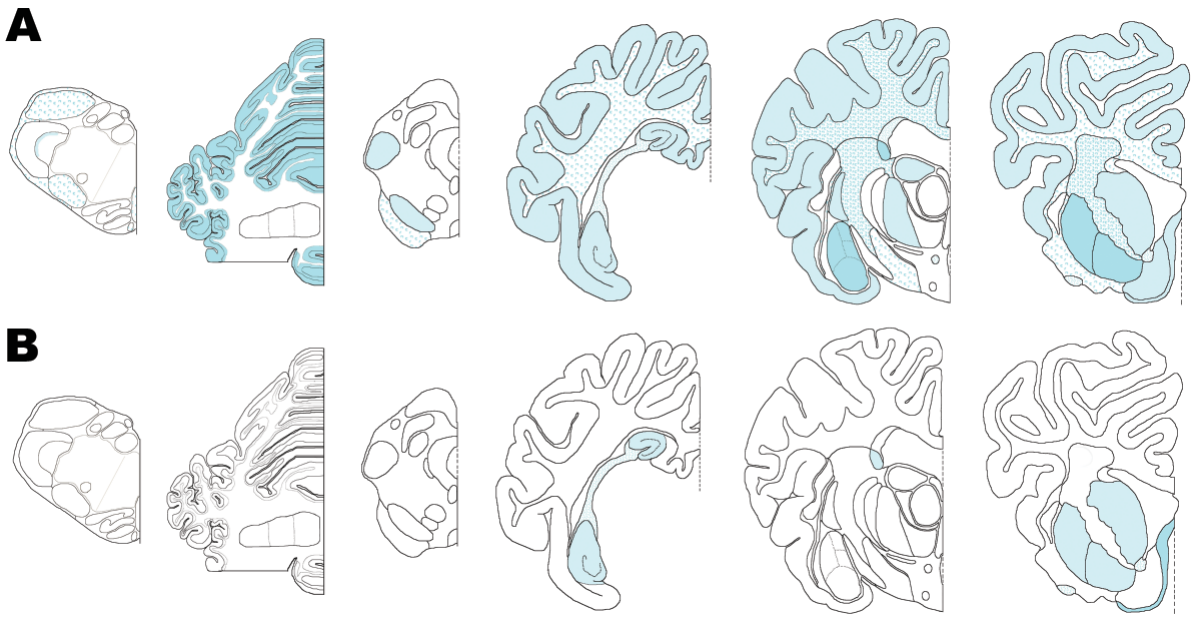
Distribution of immunolabeling in sheep infected with scrapie. A) animal 12, B) animal 11. Animal 12 exhibits the same distribution and type of immunolabeling as seen in natural disease (*23*). In animal 11, immunolabelling was much more restricted and did not involve the cerebellum.

**Figure 3:**
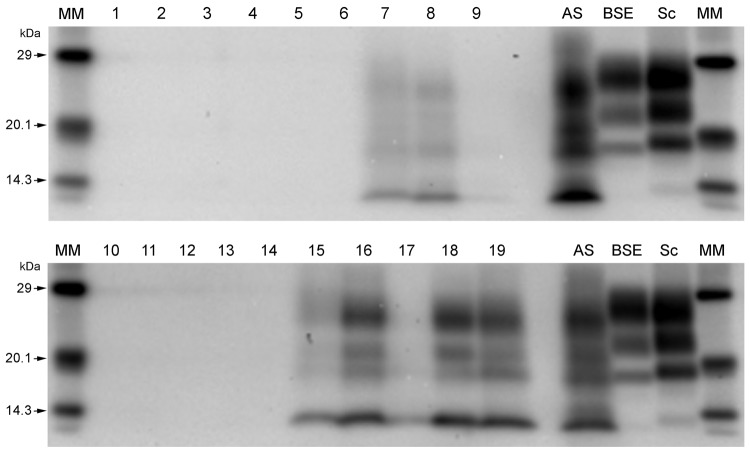
Western immunoblots showing clear atypical scrapie profiles in sheep in the following brain regions; brainstem of donor ARRa (lane 7), frontal cortex of donor ARRb (lane 8) and frontal cortex of donor AHQ (lane 19). The hippocampus and basal nuclei of recipient animal 11 (lanes 15 and 16, respectively) and cerebellum of recipient animal 12 (lane 18). No discernible signal was seen in the medulla of donor ARRb (lane 9), and only a faint profile was visible for the obex of recipient animal 12 (lane 17). Lane 1, animal 2 obex; lane 2, animal 1 obex; lane 3, animal 3 obex; lane 4, animal 4 obex; lane 5, animal 5 obex; lane 6, animal 6 obex ; lane 7, donor ARRa rostral B.stem; lane 8, donor ARRb frontal cortex; lane 9, donor ARRb caudal medulla; lane 10, animal 7 obex; lane 11, animal 8 obex; lane 12, animal 9 obex; lane 13, animal 10 obex; lane 14, case 11 obex; lane 15, animal 11 hippocampus; lane 1, animal 11 basal nuclei; lane 17, animal 12 obex; lane 18, animal 12 cerebellum; lane 19, donor AHQ frontal cortex; AS, atypical scrapie; BSE, classical bovine spongiform encephalopathy; Sc, classical scrapie; MM, molecular mass marker.

PrP^Sc^ was detected in 2 animals only, both of which were AHQ homozygotes. In animal 11, which was clinically normal, PrP^Sc^ was observed in the caudal thalamus, restricted to the lamina medullaris externa, in the caudate nucleus, the amygdala, and external capsule, and minimal labeling was found in the basal and septal nuclei. Positive immunolabeling (not shown) was seen in the hippocampus, together with extensive incidental “thready” staining (*29*). White matter labeling was found in the olfactory tract and rostral commissure (Figure 2). No PrP^Sc^ was detected in the medulla or cerebellum, the areas currently used for statutory surveillance purposes. In animal 12, PrP^Sc^ was distributed widely throughout the brain and was detectable by IHC (Figure 2), ELISA, and Western blot (Figure 3). Widespread white matter labeling and mild-to-moderate granular labeling were found, consistent with that described for natural cases of atypical scrapie (*24*) (Figure 2). No evidence of PrP^Sc^ accumulation was found in any of the examined tissues from the other animals. Western blot profiles of PrP^Sc^ from these animals were compatible with those of animals with of atypical scrapie (Figure 3).

In addition to the tissues listed in the Table, the jejunum, lateral retropharyngeal lymph node, respiratory epithelium, triceps muscle, cranial cervical ganglion, nodose ganglion, facial nerve, trigeminal ganglion, and sciatic nerve have all been screened by IHC in the PrP^Sc^-positive animals. No evidence of PrP^Sc^ accumulation outside the brain has been identified in these animals.

Bioassays of cerebellum, spleen, distal ileum, and of tissues with detectable PrP^Sc^ are still ongoing. To date, the cerebellum sample from animal 11 and the distal ileum samples from animal 12 have resulted in PrP^Sc^-positive mice, although no PrP^Sc^ was detected in those tissues (Table A1). When assays are complete, the lesion profiles obtained in the mice show that the biological profile of the experimental animals is the same as that of the donor, and that infectivity detected in the peripheral tissues has the same biologic signature as that in the brain (Figure 1).

## Discussion

This study is still ongoing and will not be completed until 2012. However, the current interim report documents the successful oral transmission of atypical scrapie, confirms that the disease phenotype is retained following transmission by this route in AHQ/AHQ sheep, and indicates that infectivity can be demonstrated in the gut in the absence of detectable PrP^Sc^ at least as early as 12 months after exposure.

One sheep (animal 12) culled at 24 months post inoculation displayed abnormalities in behavior and movement suggestive of atypical scrapie. Signs like ataxia with head tremor and circling have been described in experimental (*19*) and natural (*3**,**30*) disease, which was attributed to lesions in the cerebellum and forebrain, respectively, corresponding with PrP^Sc^ accumulation in these areas (*20**,**24*).

By contrast, animal 11, which had confirmed atypical scrapie based on postmortem tests, was considered clinically normal. The less severe and limited PrP^Sc^ accumulation in the brain of this sheep than in animal 12 may explain the absence of clinical abnormalities, which is supported by our findings in goats with scrapie in which more extensive PrP^Sc^ accumulation in the brain was usually associated with a more severe clinical disease (*25*).

Although all TSEs are transmissible after intracerebral challenge to a susceptible host, only some are infectious under natural conditions. Therefore, it was important from a pathogenesis and disease control perspective to establish whether or not oral transmission can be successful. However, the challenge model in this study exposed animals as neonates, when the esophageal groove is operational and the lambs are physiologically monogastric. Exposure of 3-month-old ruminating animals to similar amounts of positive brain by the oral route have so far not resulted in any clinical disease, with all animals still alive >1,500 days post challenge (M.M. Simmons, unpub. data), but most natural cases have been recorded in animals older than this, so these animals may still progress to disease in the next few years. Since this challenge study in older animals has no time-kill component, and no losses caused by unrelated disease have occurred, whether any of these sheep are in a preclinical phase of disease is unknown. Unfortunately, the absence of detectable PrP^Sc^ in lymphoreticular tissues of sheep with atypical scrapie precludes the use of biopsies to ascertain early infection in these animals.

Transmission may be more efficient in newborn animals; the incubation periods of sheep orally infected with classical scrapie were significantly shorter in sheep challenged at 14 days of age than those challenged at 6 months of age (*31*). If, however, oral transmission is only effective in such young animals, then field exposure would most likely have to be through milk, which is known to be a highly effective route of transmission for classical scrapie (*32*). No data are currently available on the potential infectivity of milk from animals with atypical scrapie.

Successful oral transmission also raises questions regarding the pathogenesis of this form of disease. There must be passage of the infectious agent from the alimentary canal to the brain through one of several possible routes, most likely those that have been suggested and discussed in detail for other TSEs, for example, retrograde neuronal transportation either directly (*33**–**35*) or through lymphoid structures or hematogenously (*36*). Infectivity in the absence of readily demonstrable PrP^Sc^ has been reported (*37**–**39*), and although the mouse bioassay may detect evidence of disease in other tissues, these data may not be available for at least another 2 years. More protease-sensitive forms of PrP^Sc^ may be broken down more efficiently within cells and thus do not accumulate in peripheral tissues (*19*), enabling atypical PrP^Sc^ to transit the digestive tract and disseminate through other systems in small amounts before accumulating detectably in the central nervous system.

Although we do not have epidemiologic evidence that supports the efficient spread of disease in the field, these data imply that disease is potentially transmissible under field situations and that spread through animal feed may be possible if the current feed restrictions were to be relaxed. Additionally, almost no data are available on the potential for atypical scrapie to transmit to other food animal species, certainly by the oral route. However, work with transgenic mice has demonstrated the potential susceptibility of pigs, with the disturbing finding that the biochemical properties of the resulting PrP^Sc^ have changed on transmission (*40*). The implications of this observation for subsequent transmission and host target range are currently unknown.

How reassuring is this absence of detectable PrP^Sc^ from a public health perspective? The bioassays performed in this study are not titrations, so the infectious load of the positive gut tissues cannot be quantified, although infectivity has been shown unequivocally. No experimental data are currently available on the zoonotic potential of atypical scrapie, either through experimental challenge of humanized mice or any meaningful epidemiologic correlation with human forms of TSE. However, the detection of infectivity in the distal ileum of animals as young as 12 months, in which all the tissues tested were negative for PrP^Sc^ by the currently available screening and confirmatory diagnostic tests, indicates that the diagnostic sensitivity of current surveillance methods is suboptimal for detecting atypical scrapie and that potentially infectious material may be able to pass into the human food chain undetected.

## Appendix

**Appendix:** Table. Immunohistochemical/ELISA results for sheep infected with scrapie for each tissue*

Genotype	ARR/ARR								AHQ/AHQ						
Months postinoculation	12				24				12				24		
Donor inoculum	ARRa	ARRa	ARRa		ARRa	ARRa	ARRb		AHQ	AHQ	AHQ		AHQ	AHQ	AHQ
Animal no.	1	2	3		4	5	6		7	8	9		10	11	12
Clinical status	Norm	Norm	Inc		Norm	Norm	Inc		Norm	Norm	Norm		Inc	Norm	Suspect
Obex	–/NT	–/NT	–/NT		–/NT	–/NT	–/NT		–/NT	–/NT	–/NT		–/NT	–/NT	–/NT
Cerebellum†	−/−†	−/−	−/−		−/−	−/−	−/−		−/−	−/−	−/−		−/−	−/−‡	+/+‡
Rostral medulla	–/NT	–/NT	–/NT		–/NT	–/NT	–/NT		–/NT	–/NT	–/NT		–/NT	–/NT	–/NT
Caudal midbrain	–/NT	–/NT	–/NT		–/NT	–/NT	–/NT		–/NT	–/NT	–/NT		–/NT	–/NT	–/NT
Rostral midbrain	–/NT	–/NT	–/NT		–/NT	–/NT	–/NT		–/NT	–/NT	–/NT		–/NT	–/NT	–/NT
Thalamus	−/−	−/−	−/−		−/−	−/−	−/−		−/−	−/−	−/−		−/−	+/−‡§	+/+
Occipital cortex	–/NT	–/NT	–/NT		–/NT	–/NT	–/NT		–/NT	–/NT	–/NT		–/NT	–/NT	–/NT
Parietal cortex	–/NT	–/NT	–/NT		–/NT	–/NT	–/NT		–/NT	–/NT	–/NT		–/NT	–/NT	–/NT
Frontal cortex	−/−	−/−	−/−		−/−	−/−	−/−		−/−	−/−	−/−		−/−	+/+‡	+/+
Spinal cord C5	–/NT	–/NT	–/NT		–/NT	–/NT	–/NT		–/NT	–/NT	–/NT		–/NT	–/NT	–/NT
Spinal cord T1	–/NT	–/NT	–/NT		–/NT	–/NT	–/NT		–/NT	–/NT	–/NT		–/NT	–/NT	–/NT
Spinal cord T6	–/NT	–/NT	–/NT		–/NT	–/NT	–/NT		–/NT	–/NT	–/NT		–/NT	–/NT	–/NT
Spinal cord T12	−/−	−/−	–/ITT		−/−	−/−	−/−		−/−	−/−	−/−		−/−	−/−	−/−
Spinal cord L5–6	–/NT	–/NT	–/NT		–/NT	–/NT	–/NT		–/NT	–/NT	–/NT		–/NT	–/NT	–/NT
Palatine tonsil	–/ITT	–/ITT	–/ITT		–/ITT	–/ITT	–/ITT		–/ITT	–/ITT	–/ITT		–/ITT	–/ITT	–/ITT
Spleen†	−/−	−/−	−/−		−/−	−/−	−/−		−/−	−/−	−/−		−/−	−/−	−/−
Mesenteric lymph node	−/−	−/−	−/−		−/−	−/−	−/−		−/−	−/−	−/−		−/−	−/−	−/−
Distal ileum†	−/−	−/−	−/−		−/−	−/−	−/−		−/−	−/−‡	−/−‡		−/−	−/−	−/−‡
RAMALT	–/NT	–/NT	–/NT		–/NT	–/NT	–/NT		–/NT	–/NT	–/NT		–/NT	–/NT	–/NT
Coeliaco-mesenteric plexus	–/NT	–/NT	–/NT		–/NT	–/NT	–/NT		–/NT	–/NT	ITT/NT		–/NT	–/NT	–/NT
Extraocular muscles	–/ITT	–/ITT	–/ITT		−/−	–/ITT	−/−		–/ITT	−/−	−/−		−/−	−/−	−/−
Medial retropharyngeal lymph node	NT/–	NT/–	NT/–		NT/–	NT/–	NT/–		NT/–	NT/–	NT/–		NT/–	−/−	−/−
Submandibular lymph node	NT/ITT	NT/ITT	NT/ITT		NT/ITT	NT/–	NT/–		NT/–	NT/ITT	NT/ITT		NT/–	/ITT	−/−
Submandibular salivary gland	NT/–	NT/–	NT/–		NT/–	NT/–	NT/–		NT/–	NT/–	NT/–		NT/–	−/−	−/−
Cervical thymus	NT/–	NT/–	NT/–		NT/–	NT/–	NT/–		NT/–	NT/–	NT/–		NT/–	−/−	−/−
Prescapular lymph node	NT/–	NT/–	NT/–		NT/–	NT/–	NT/–		NT/–	NT/–	NT/–		NT/–	−/−	−/−
Kidney	NT/–	NT/–	NT/–		NT/–	NT/–	NT/–		NT/–	NT/–	NT/–		NT/–	−/−	−/−
Liver	NT/–	NT/–	NT/–		NT/–	NT/–	NT/–		NT/–	NT/–	NT/–		NT/–	−/−	−/−
Buffy coat	NT/–	NT/–	NT/–		NT/ITT	NT/ITT	NT/ITT		NT/–	NT/–	NT/–		NT/ITT	NT/ITT	NT/ITT

*ARR/ARR, Prp genotype of 6 Poll Dorset lambs; AHQ/AHQ, PrP genotype of 6 Cheviot lambs; mpi, months post inoculation; Norm, normal; Inc, inconclusive; Suspect, suspected; NT, not tested; ITT, insufficient tissue to test; RAMALT, recto-anal mucosa–associated lymphoid tissue; PrP^Sc^ or protease-resistant form present (+) or absent (–). †Tissues being investigated by bioassay in Tg338 mice. ‡Tissues are positive in bioassay. §High negative result in ELISA.
